# Sodium-fluoride PET-CT for the non-invasive evaluation of coronary plaques in symptomatic patients with coronary artery disease: a cross-correlation study with intravascular ultrasound

**DOI:** 10.1007/s00259-018-4122-0

**Published:** 2018-08-31

**Authors:** Li Li, Xiang Li, Yongping Jia, Jiamao Fan, Huifeng Wang, Chunyu Fan, Lei Wu, Xincheng Si, Xinzhong Hao, Ping Wu, Min Yan, Ruonan Wang, Guang Hu, Jianzhong Liu, Zhifang Wu, Marcus Hacker, Sijin Li

**Affiliations:** 1 Department of Nuclear Medicine, The First Hospital of Shanxi Medical University, Molecular Imaging Precision Medical Collaborative Innovation Center, Taiyuan, 030001 Shanxi Province China; 2 Division of Nuclear Medicine, Department of Biomedical Imaging and Image-guided Therapy, Medical University of Vienna, Vienna, Austria; 3 Department of Cardiology, The First Hospital of Shanxi Medical University, Taiyuan, Shanxi China; 4 Department of Cardiology, The Fourth People’s Hospital of Linfen, Linfen, Shanxi China; 5 Department of Cardiology, Taigang General Hospital, Shanxi Medical University, Taiyuan, Shanxi China

**Keywords:** Coronary plaque, ^18^F-sodium fluoride, Positron emission tomography, Intravascular ultrasound

## Abstract

**Objectives:**

The aim of this study was to evaluate the ^18^F-sodium fluoride (^18^F-NaF) coronary uptake compared to coronary intravascular ultrasound (IVUS) in patients with symptomatic coronary artery disease.

**Background:**

^18^F-NaF PET enables the assessment of vascular osteogenesis by interaction with surface hydroxyapatite, while IVUS enables both identification and quantification of intra-plaque components.

**Methods:**

Forty-four patients with symptomatic coronary artery disease were included in this prospective controlled trial, 32 of them (30 patients with unstable angina and 2 patients with stable angina), representing the final study cohort, got additional IVUS. All patients underwent cardiac ^18^F-NaF PET/CT and IVUS within 2 days. ^18^F-NaF maximum tissue-to-blood ratios (TBR_max_) were calculated for 69 coronary plaques and correlated with IVUS plaque classification.

**Results:**

Significantly increased ^18^F-NaF uptake ratios were observed in fibrocalcific lesions (meanTBR_max_ = 1.42 ± 0.28), thin-cap atheroma with spotty calcifications (meanTBR_max_ = 1.32 ± 0.23), and thick-cap mixed atheroma (meanTBR_max_ = 1.28 ± 0.38), while fibrotic plaques showed no increased uptake (meanTBR_max_ = 0.96 ± 0.18). The ^18^F-NaF uptake ratio was consistently higher in atherosclerotic lesions with severe calcification (meanTBR_max_ = 1.34 ± 0.22). The regional ^18^F-NaF uptake was most likely localized in the border region of intensive calcification. Coronary lesions with positive ^18^F-NaF uptake showed some increased high-risk anatomical features on IVUS in comparison to ^18^F-NaF negative plaques. It included a significant severe plaque burden (70.1 ± 13.8 vs. 61.0 ± 13.8, *p* = 0.01) and positive remodeling index (1.03 ± 0.08 vs. 0.99 ± 0.07, *p* = 0.05), as well as a higher percentage of necrotic tissue (37.6 ± 13.3 vs. 29.3 ± 15.7, *p* = 0.02) in positive ^18^F-NaF lesions.

**Conclusions:**

^18^F-NaF coronary uptake may provide a molecular insight for the characterization of coronary atherosclerotic lesions. Specific regional uptake is needed to be determined by histology.

**Electronic supplementary material:**

The online version of this article (10.1007/s00259-018-4122-0) contains supplementary material, which is available to authorized users.

## Introduction

The progression of atherosclerosis involves complex pathophysiology, mainly including cell migration, apoptosis, inflammation, and osteogenesis, which drive calcification-prone lesions to become atherosclerotic plaques [1, 2].

Calcification is a remarkable biomarker of coronary atherosclerotic plaque burden, and coronary calcification on computed tomography (CT) may be a predictor of clinical disease [3]. Additional evidence has shown that the presence of intra-plaque microcalcification or spotty calcification, along with thin, fibrous caps, is associated with a high risk for plaque rupture, which can lead to acute thrombosis and even fatal cardiac events [4–6].

As therapeutic strategy to prevent heart attacks, the selective reorganization of vulnerable atherosclerotic lesions has become a major task in cardiovascular research. The vulnerability of coronary plaques is conventionally assessed using the criteria of luminal stenosis, fibrous cap, lipid core, and severity of calcification by anatomical imaging, such as coronary CT angiography [7], cardiac magnetic resonance imaging (MRI) [8], and non-contrast CT [9]. Noninvasive low-dose electron-beam CT is a conventional imaging technique to quantify calcium in the coronary arteries, and CT-derived coronary calcium scores have been commonly used for measurement of arterial macrocalcification. However, although the extent of coronary calcification could be determined, accurate quantification of various plaque components on conventional CT is still challenging due to limited spatial resolution for microcalcification.

The composition of coronary plaques shows substantial variability, and it would be extremely useful to improve the differentiation of the various components within plaques. Catheter-based intravascular ultrasound (IVUS) was introduced for this purpose, and demonstrated a greater sensitivity for plaque tissue detection, which did offer insights into plaque components but in an inadequacy of characterization for intraplaque activity [10].

Currently, the early identification of patients at high-risk for plaque rupture has become a primary goal in the prevention of a cardiac event. Sodium fluoride (^18^F-NaF) with an affinity to hydroxyapatite was introduced as a positron emission tomography (PET) tracer for the ongoing evaluation of vascular osteogenesis in atherosclerotic plaques [11, 12].

This prospective multi-modality study assessed the inter-relationship between the severity of coronary calcification (CT calcium score), osteogenesis activity (^18^F-NaF PET uptake ratio) and the coronary plaque composition by IVUS. The major aim was to determine the feasibility the coronary ^18^F-NaF PET for assessing atherosclerotic lesions of patients with cardiovascular disease, associated with intraplaque components, which could provide insight into coronary artery disease.

## Methods

### Study participants

**Fig. 1:**
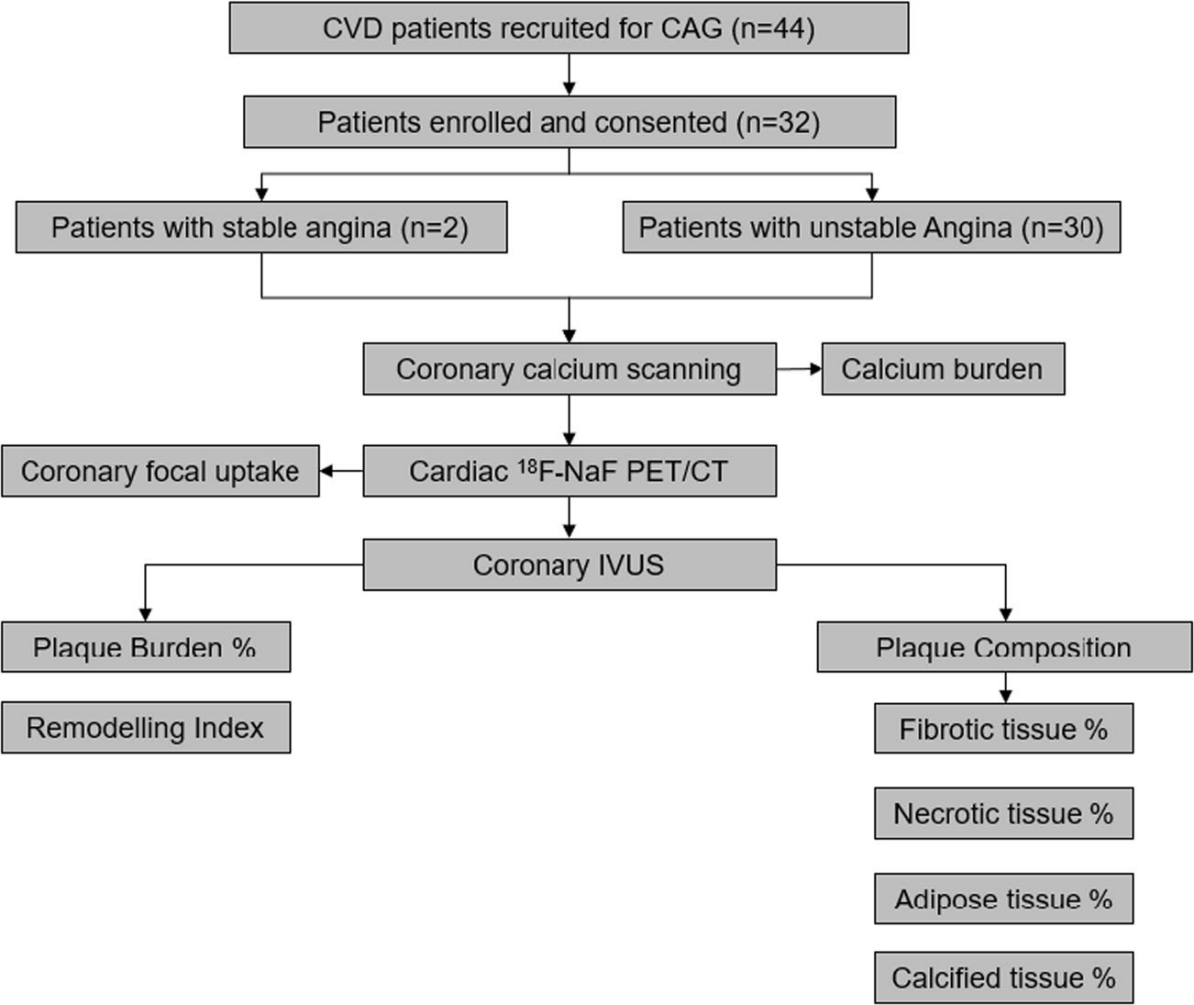
The flowcharts of study. CAD: cardiovascular disease, CAG: coronary angiography, PET/CT: positron emission tomography/computed tomography, IVUS: intravascular ultrasound

At baseline clinical assessment, 44 patients with symptomatic coronary artery disease who underwent coronary angiogram for the purpose of guiding implantation of vascular scaffolds were prospectively recruited between November 2015 and August 2017. The Institutional Review Board approved this prospective controlled study and patients provided written, informed consent. Twelve patients with renal failure, vasculitis, myocarditis, and metastatic malignancy were excluded. So that finally 32 patients (22 men, 10 women) could be included in this prospective cross-sectional trial (Fig. 1).

### Imaging acquisition and analysis

#### Invasive coronary angiography and IVUS

Patients (*n* = 44) with unstable and stable angina underwent conventional coronary angiography. Standard IVUS was performed at coronary artery stenosis which were guided by coronary angiography in 32 patients [13, 14]. The catheter was positioned distal to the lesions and virtual histology data were acquired with a commercially available imaging system (Atlantis, Boston Scientific, iLabTM ultrasound imaging system) incorporating 40-MHz transducer-tip catheter (diameter 9 mm) and motorized pullback at 0.5 mm/s. Quantitative IVUS measurements were performed at the same lesions and both proximal and distal reference were determined by an experienced IVUS analyst.

The cross-tissue, color-coded maps were constructed by IVUS radiofrequency signal to characterize plaque components, mainly including fibrous tissue (green), fibrolipid (yellow), lipid necrotic core (pink), and calcium (blue). The fibrous cap thickness, lipid necrotic core, plaque burden, and remodeling parameters were recorded. Coronary plaque lesions (*n* = 69) assessed by in vivo IVUS data were classified by an experienced interventional cardiologist, who was blinded to PET/CT scans, as type1, thick-cap fibroatheroma with dense necrotic tissue; type 2, thin-cap fibroatheroma with dense necrotic tissue; Type 3, fibrotic plaque poor necrotic tissue; and Type 4, fibrocalcific plaque with intensive calcification [15, 16].

#### ^18^F-NaF PET-CT and blood serum tests

Electrocardiograph-gated PET-CT imaging was performed prior to IVUS in patients scheduled for percutaneous coronary intervention within 2 days. All patients underwent electrocardiography (ECG)-gated ^18^F-NaF PET-CT imaging on a Discovery VCT combined PET-CT system (GE Healthcare, USA). Prior to the PET scan, using the combined GE Discovery VCT PET-CT scanner with an integrated 64-slice CT scanner, a low-dose CT (120 kV, 50 mA) with attenuation correction was acquired for anatomical co-registration of ^18^F-NaF uptake. The radiochemical purity of ^18^F-NaF reached 99%, which was tested by the manufacturer (Bioscan Corporation, USA). After 60 min of tracer circulation, all patients underwent 10-min static ^18^F-NaF PET scanning in one bed position, covering the heart and the aortic arch.

All PET/CT scans were assessed by two experienced nuclear physicians, based on coronary anatomical imaging determined by CT scans; two-dimensional regions of interest (ROIs) with a diameter of 1–2 mm were selected manually to target the coronary vessels, including the left main coronary artery (LMCA), the left anterior descending (LAD), the left circumflex (LCx), and the right coronary artery (RCA). Maximum standardized uptake values (SUV_max_) were derived from ROIs. Maximum tissue-to-blood (TBR_max_) values were obtained by correcting SUVmax values with mean of blood pool activity (SUV_blood_) from the three ROIs placed in the lumen of the superior vena cava [17, 18]. Self-defined cut-off values of TBR_max_ = 1.25 were used to categorize coronary lesions (*n* = 69) according to 1.25 times increased uptake ratio in relation to the blood pool (TBR_mean_). ^18^F-NaF negative atherosclerotic lesions thus had a TBR_max_ < 1.25 and positive atherosclerotic lesions had a TBR_max_ ≥ 1.25.

We used a standard commercial biochemical test to measure serum cholesterol, low-density lipoprotein (LDL), high-density lipoprotein (HDL), and high-sensitivity C-reactive protein (hsCRP).

#### Coronary calcium burden from electron beam CT

All patients (*n* = 32) underwent calcium scanning of the four coronary arteries (*n* = 128)—the left main coronary artery (LMCA), the left anterior descending artery (LAD), the left circumflex artery (LCx), and the right coronary artery (RCA)—(tube voltage:120 kV, current:200mAs) with a 3.0-mm slice thickness and reconstructed with the B36f kernel. Volume analysis software (GE Health Care, Waukesha, WI, USA) was used to discern calcium from other tissues, with a threshold of 130 Hounsfield units. Agatston calcium scores (AS), calcium mass, and calcium volume were determined automatically. All lesions were distributed into five subgroups based on AS: group 1 (no calcification (AS = 0, *n* = 46); group 2, minimal calcification (0 < AS≤10, *n* = 22); group 3, mild calcification (10 < AS≤100, *n* = 27); group 4, moderate calcification (100 < AS≤400, *n* = 24); and group 5, severe calcification (AS>400, *n* = 9).

#### Correlation of imaging methods

For image correlation due to different image formats of the applied methods, IVUS results were used as reference for anatomical localization of coronary vessels and segments (distal, proximal or mid). PET/CT ROIs were then drawn and evaluated on the corresponding regions in the axial views.

#### Statistics

All continuous data were recorded as mean ± SD. Parametric data were statistically compared using one-way ANOVA. The Mann-Whitney U test was used for the non-parametric variables in the group comparison. The Pearson’s linear correlation coefficient was used to assess the correlations between mean TBR_max_ (mTBR_max_) from each patient and corresponding biochemical data including cholesterol, LDL, HDL, and hsCRP from a serum test. Multivariate linear regression analysis was also performed to determine the independent calcium factors, including coronary calcium mass and calcium volume. We used a two-way random intraobserver observation for ICCs greater than 0.9 as an indicator of excellent reproducibility between two experienced readers. All statistical analyses were performed in SPSS v. 19 (SPSS Inc., Chicago, IL). *P*-values <0.05 were considered statistically significant.

Intra-class correlation coefficients (ICCs) with 95% confidence intervals were calculated to test inter- and intra-observer agreement for the TBR values. Two-way random ICC values of more than 0.8 were accepted as a measure of excellent reproducibility.

## Results

### Patients

Detailed clinical baseline data including cardiovascular symptoms, prior coronary intervention, cardiac events, as well as common risk factors for cardiovascular diseases, are presented in Table 1. Additional data about medication and serum biochemical measurement from a venous blood sample are also listed in Table 1.

**Table 1:** Patient characteristics

Characteristic	Unstable angina (N = 30)	Stable angina (N = 2)
Men, *n* (%)	20(67)	2(100)
Age, mean (SD)	57(8)	65(2)
BMI (kg/m^2^), mean (SD)	25.0(3.1)	26.3(0.7)
HR (per min), mean (SD)	69.2(8.7)	88.0(14.1)
SBP (mm Hg), mean (SD)	137.4(16.4)	137.5(6.4)
DBP (mm Hg), mean (SD)	82.8(11.2)	76.5(12.0)
History of cardiovascular diseases, n (%)		
Previous MI	2(7)	0
Previous CVA/TIA	6(20)	0
Risk factors, *n* (%)		
Smoking (ex or current)	19(63)	1(50)
Diabetes	10(33)	2(100)
Hypertension	17(57)	2(100)
Hypercholesterolemia	8(27)	1(50)
Medications, *n* (%)		
Aspirin	13(43)	1(50)
Clopidogrel	7(23)	0
Statin	13(43)	1(50)
β blocker	4(13)	1(50)
ACEI/ARB	4(13)	0
Calcium channel blockers	11(37)	1(50)
Oral nitrates	2(7)	0
Other anti-hypertensive	2(7)	0
Insulin	5(17)	1(50)
Melbine/Acarbose/Sulfonylureas	4(13)	0
Serum biochemistry, mean (SD)		
Cholesterol (mmol/L)	4.3(1.3)	4.3(2.2)
LDL cholesterol (mmol/L)	2.6(1.1)	3.0(2.0)
HDL cholesterol (mmol/L),	1.0(0.3)	1.0(0.2)
Triglycerides (mmol/L)	2.2(2.0)	0.9(0.5)
Fasting blood-glucose (mmol/L)	6.4(3.0)	10.6(3.4)
Hemoglobin A1c (%)	5.9(1.8)	7.6(0.5)
hemoglobin (G/L)	141(13)	142(13)
Creatinine (μmol/L)	71.4(15.5)	50.9(10.1)
Blood urea nitrogen (μmol/L)	71.4(15.5)	3.4(0.9)
hsCRP (mg/L)	4.9(1.6)	7.2(0.5)
Coronary calcifications		
AJ-130 (Agatston units)	498.1(688.8)	387.0(449.7)
Mass (mg)	74.4(109.5)	47.0(62.2)
Volume (mm^3^)	203.8(254.7)	143.5(170.4)

BMI: Body-mass index, HR: Heart rate, SBP: Systolic blood pressure, DBP: Diastolic blood pressure, MI: Myocardial infraction, CVA: Cerebrovascular accident, TIA: transient ischemic attack, ACEI: Angiotensin-converting enzyme inhibitor, ARB: Angiotensin receptor blocker, HDL: High-density lipoprotein, LDL: Low-density lipoprotein, CAC: Coronary artery calcium, hsCRP: high-sensitivity C-reactive protein

### Lesion characterization of coronary plaques

**Fig. 2:**
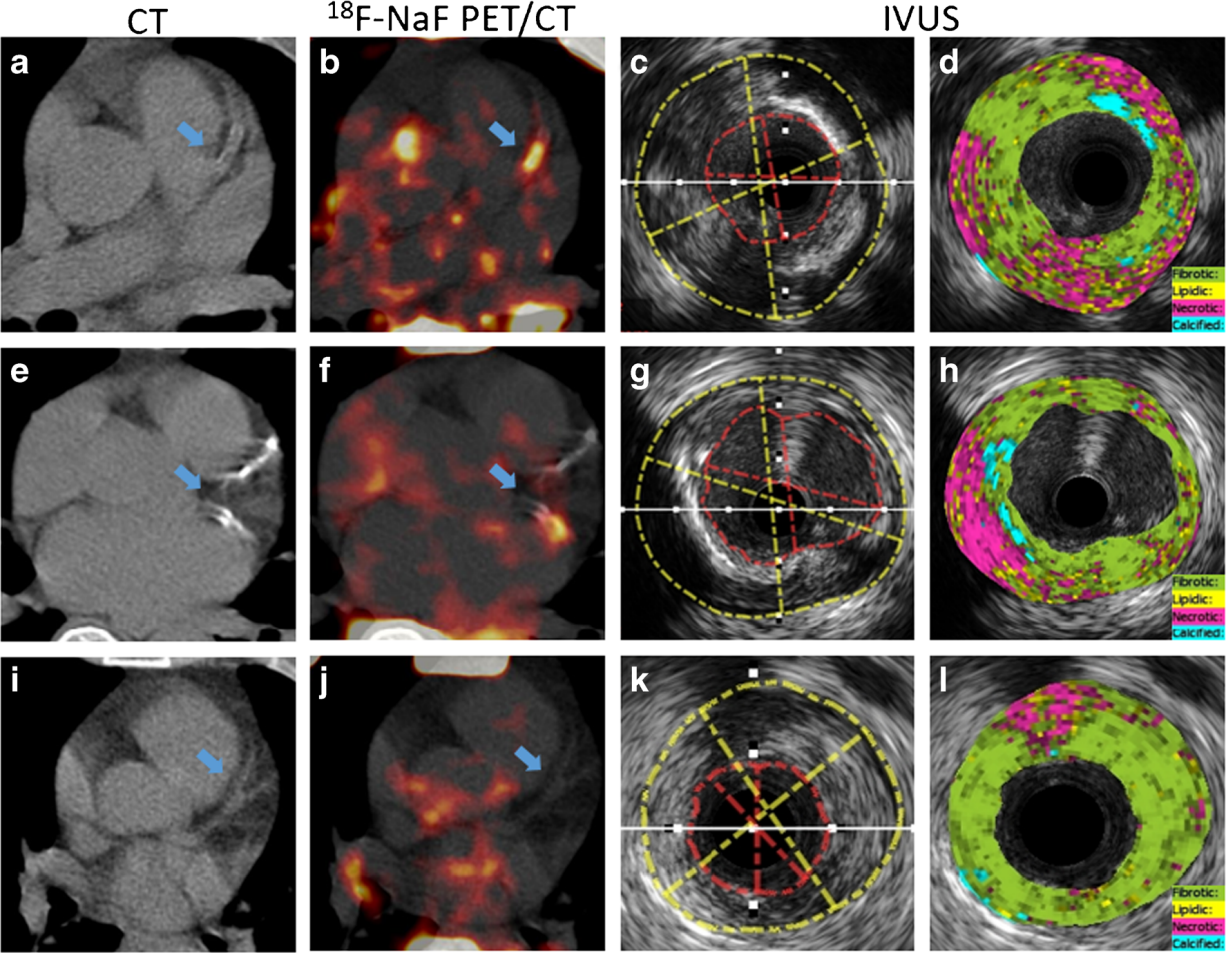
Representative images of PET/CT and IVUS from three patients with unstable angina. Increased ^18^F-NaF focal uptake was seen at atheromatous lesions with spotty calcification (**a–d**) in segments of the LAD, and thin-cap fibrocalcified lesions with a large necrotic core (**e–h**) in segments of the LCX. But, there was no uptake in fibrotic lesions (**i–l**) in segments of the LAD

**Fig. 3:**
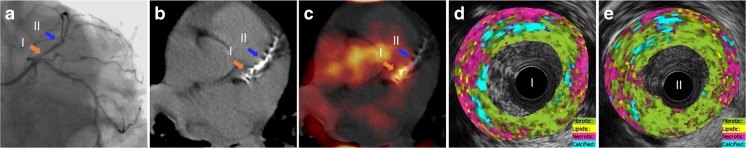
Regional coronary uptake of ^18^F-NaF. A 64-year-old woman with unstable angina. (**a**), X-ray coronary angiogram of showed severe coronary stenosis at proximal (I) and mid (II) segments of LAD; Cardiac CT (**b**) and ^18^F-NaF PET/CT (**c**) showed regional distribution of ^18^F-NaF uptake within an atheromatous lesion. Representative LAD lesions showed distinct tracer accumulation along a severely fibrocalcific lesion. Prominent focal ^18^F-NaF uptake were observed at coronary segment I verse absent focal uptake at segment II. Anatomical IVUS indicate the presence of fibroatheromatous lesion with prominently increased necrotic (35% vs. 23%) and calcified tissue (10% vs. 6%) also a slightly higher proportion of lipidic tissue (9% vs.8%), but decreased fibrotic tissue (46 vs 62%) in segment I (**d**) in comparison to segment II (**e**)

**Fig. 4:**
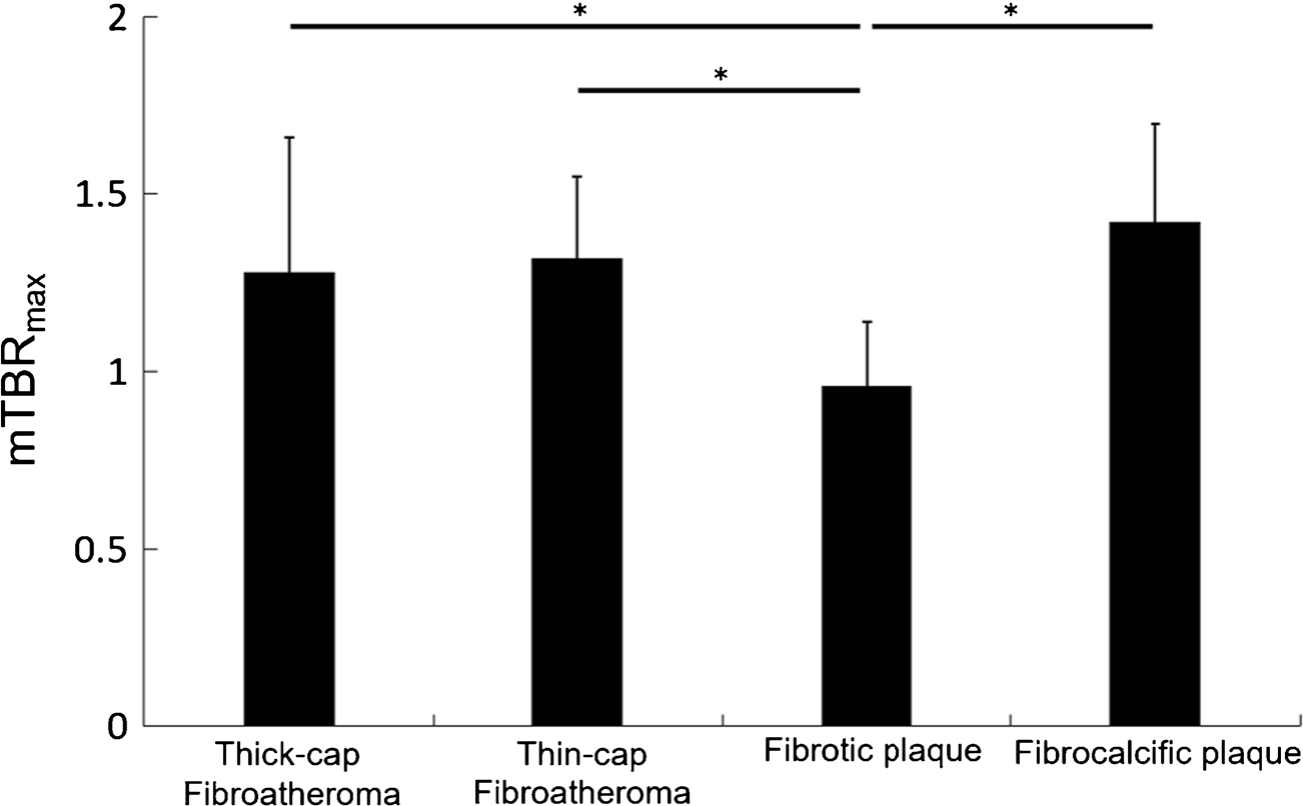
Statistical ^18^F-NaF uptake ratios (mTBR_max_) distribution within different types of atherosclerotic lesions (*n* = 69). Significantly increased uptake was observed at thick-cap (*n* = 21) and thin-cap fibroatheromas (*n* = 22) with a large necrotic core, as well as in fibrocalcified (*n* = 8) plaques compared to fibrotic plaques (*n* = 18) with less lipid necrotic tissue. mTBR_max_: mean of TBR_max_ derived from all lesions in each defined group. *p value <0.05

A total of 69 coronary atherosclerotic lesions were analyzed from 32 patients who underwent IVUS for tissue characterization of coronary plaques, including nonculprit thick-cap mixed atheromatous (*n* = 21), fibrotic atheromatous lesions (*n* = 18), calcified atheroma lesions (*n* = 8), and culprit thin-cap atheromatous lesions (*n* = 22) with spotty calcification. Discrepant uptake of ^18^F-NaF in atheromatous lesions with spotty calcification, thin-cap fibrocalcified lesions with a large necrotic core and fibrotic lesions are shown in Fig. 2. Significantly increased ^18^F-NaF signals were observed in fibrocalcific lesions (mTBR_max_ = 1.42 ± 0.28) (*n* = 8), as well as thin-cap atheroma with spotty calcifications (mTBR_max_ = 1.32 ± 0.23) (*n* = 22) and thick-cap mix atheroma (mTBR_max_ = 1.29 ± 0.38) (*n* = 21). Interestingly, ^18^F-NaF mTBR_max_ was significantly lower in fibrous plaque (mTBR_max_ = 0.97 ± 0.19) (*n* = 18) (Figs. 3 and 4). Thirty-eight lesions showed a positive ^18^F-NaF uptake with a TBR_mean_ ≥ 1.25, while 31 lesions with a TBR_mean_ < 1.25 were rated negative by definition. Inter- and intra-observer ICCs for TBR_max_ were 0.97 (95% CI, 0.95–0.99) and 0.92 (95% CI, 0.90–0.97), respectively.

The correlation between ^18^F-NaF uptake value and anatomical features of vulnerable plaque was determined. Compared to negative ^18^F-NaF lesions, a significant severe plaque burden (70.1 ± 13.8 vs. 61.0 ± 13.8, *p* = 0.01) and remodeling (1.03 ± 0.08 vs. 0.99 ± 0.07, *p* = 0.05), as well as a higher percentage of necrotic tissue (37.6 ± 13.3 vs. 29.3 ± 15.7, *p* = 0.02) were detected in positive ^18^F-NaF lesions, but with significantly lower intraplaque fibrotic tissue (46.4 ± 15.6 vs. 56.3 ± 18.7, *p* = 0.02). No significantly distinguished adipose (12.5 ± 4.2 vs. 12.0 ± 4.4, *p* = 0.60) and calcified components (3.4 ± 2.9 vs. 2.5 ± 3.1, *p* = 0.21) were detected between negative and positive ^18^F-NaF lesions (Table 2)**.**

**Table 2:** Tissue components within negative ^18^F-NaF lesions and positive ^18^F-NaF lesions

Measure	Negative ^18^F-NaF lesions	Positive ^18^F-NaF lesions	p value
Remodeling index	0.99 ± 0.07	1.03 ± 0.08	0.05
Burden (%)	61.0 ± 13.8	70.1 ± 13.8	0.01
Fibrotic tissue (%)	56.3 ± 18.7	46.4 ± 15.6	0.02
Necrotic tissue (%)	29.3 ± 15.7	37.6 ± 13.3	0.02
Adipose tissue (%)	12.0 ± 4.4	12.5 ± 4.2	0.60
Calcified tissue (%)	2.5 ± 3.1	3.4 ± 2.9	0.21

In the sub-group assessment of defined patient populations based on Agatston calcium scores, the ^18^F-NaF uptake ratio derived from 128 lesions was consistently higher in atherosclerotic lesions with severe calcification (*n* = 8, mTBR_max_ = 1.38 ± 0.19) compared to non-calcified coronary segments (mTBR_max_ = 1.16 ± 0.31, AS = 0, *n* = 46), minimal calcification (mTBR_max_ = 1.23 ± 0.48, 0 < AS≤10,*n* = 22), mild calcification (mTBR_max_ = 1.21 ± 0.27, 10 < AS≤100, *n* = 27), and moderate calcification (mTBR_max_ = 1.20 ± 0.25, 100 < AS≤400, *n* = 25). However, no significant inter-group difference was observed.

### Serum biochemistry

**Fig. 5:**
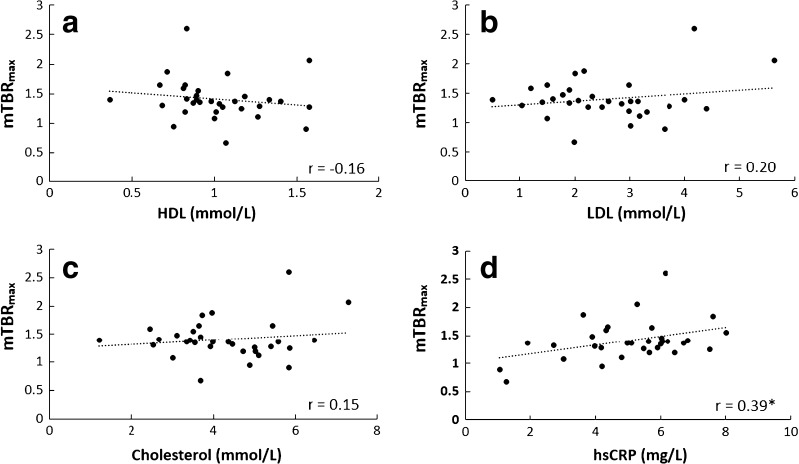
Graphs show correlation of coronary ^18^F-NaF mTRB_max_ versus clinical serum profiles. Cardiovascular biomarkers, including HDL, LDL, cholesterol level, and hsCRP level were assessed. mTBRmax: mean of TBR_max_ from all ROIs placed in individual patient. *p value <0.05

^18^F-NaF uptake ratios in relation to serum biomarkers were assessed to explore how vascular osteogenesis mechanisms are linked to underlying pathology. The hsCRP (mg/L) (*r* = 0.39, *p* < 0.05) showed significant correlation with ^18^F-NaF mTBR_max_ within individual patients using a multivariate linear regression. However, no statistically significant linear associations were observed between mTBR_max_ and serum HDL, LDL, and cholesterol levels (Fig. 5).

## Discussion

Currently, there is a great demand for in vivo quantitative estimation of on-site biomarkers for atherosclerotic imaging [19]. An abundance of evidence shows that intra-plaque activated calcification is associated with increased vulnerability of plaques and coronary events [20]. ^18^F-NaF, interacting with picomolar hydroxyapatite, a kind of calcium salt and critical component in the progression of vascular intimal calcification [21], enables the quantification of on-site molecular osteogenesis, which is not possible by conventional CT [22]. CT has shown great potential in the quantification of arterial calcium extent based on the Agatston calcium score [23]. Consequently, CT-derived Agatston calcium scores might overestimate the hydroxyapatite amount and there might be complementary information between ^18^F-NaF PET and CT.

In the characterization of atherosclerotic lesions, prior to anatomical abnormalities observed on CT scans, ^18^F-NaF PET has recently shown great potential to identify vascular microcalcification, which was recognized as a major component during the inflammatory process in vulnerable plaques [24]. Evidential, prospective pioneering studies showed significantly increased ^18^F-NaF uptake ratios in patients with atherosclerosis compared to control patients. And, higher uptake value was detected in association with symptomatic, morphologically-risky coronary plaques [11]. Nevertheless, the indication of increased ^18^F-NaF uptake within different categorized atherosclerotic lesions is still controversial.

In a previous study of ^18^F-NaF plaque imaging, we observed that the majority of hot ^18^F-NaF lesions were detected in mildly and severely calcified lesions on low-dose CT scans in large arteries [17], with increased ^18^F-NaF uptake in the spotty calcifications as well as dense macrocalcifications. The results from the present coronary trial are in accordance with this previous study on large arteries. Interestingly, regional ^18^F-NaF uptake was most likely localized in the border region of intensive calcification where there was concurrent active inflammation. Thus, the border regional ^18^F-NaF uptake might indicate increase of calcium, and prominent intra-plaque ^18^F-NaF accumulation might predict active crystallization and accelerated progression of atherosclerosis. However, whether this active calcification drives plaque stabilization or appears due to lesion erosion needs to be determined, since osteogenic differentiation could be induced from immune cells and smooth muscle cells. A larger cohort in a prospective study has shown an inverse relationship between ^18^F-NaF arterial uptake and arterial calcification burden. Those authors demonstrated that 41% of patients with severely calcified atheroma (calcium scores >1000) rarely demonstrated a positive ^18^F-NaF uptake, but there was strong tracer accumulation at the peri-region of severe calcification [11]. They also suggested that ^18^F-NaF might indicate active calcification.

As a consequence, arterial ^18^F-NaF uptake might act as a biomarker of on-site metabolic osteogenesis related to the specific surface density of hydroxyapatite, rather than simply the intraplaque calcification burden. And, regionally increased ^18^F-NaF uptake might indicate active calcium growth occurring at the border region of severe calcification. Furthermore, this could be due to the heterogeneity of plaques and the dynamic interplay between macrophage-derived microcalcification [25] and smooth muscle cell-derived macrocalcification [26].

In this study, we observed ^18^F-NaF uptake at fibrocalcific lesions, indicating an association between the vascular ^18^F-NaF uptake and the mineralization of plaques. Moreover, ^18^F-NaF uptake was absent in several calcified coronary lesions. There are several reasons which may contribute to this. (1) There was increased partial volume effect on PET scans for small lesions. (2) The ^18^F-NaF interplay with the calcium salt of hydroxyapatite depends on the nature of the specimen, and real localized osteogenic bioactivity is still unclear. Additionally, although hydroxyapatite is a critical component in the progression of vascular intimal calcification [21], various types of calcium salts deposit within an atherosclerotic artery [27]. (3) There is increased incorporation of ^18^F-NaF with the newly formed hydroxyapatite compared to old hydroxyapatite crystals [28]; thus, the adsorption of fluoride to hydroxyapatite is discrepant between patients and lesions. (4) Activation of covalent binding between sodium fluoride and arterial hydroxyapatite components under various physiological or chemical conditions might be another alternative.

Indeed, we proposed that the anatomical composition on CT with respect to increased real-time ^18^F-NaF uptake at plaque lesions reflected underlying hypometabolism; however, the pattern of temporal evolution of osteogenesis and inflammation during plaque growth was not confirmed in prior studies, where in particular the tracer kinetics of ^18^F-NaF in arterial lesions are still unclear. Therefore, to detect the vasculature formation of hydroxyapatite over time is a major task for ^18^F-NaF coronary PET.

Using IVUS as an anatomical reference, we found the ^18^F-NaF uptake was not directly associated with arterial calcium burden, but more intensively accumulated in the atheroma with spotty calcification and calcified fibroatheroma.

In our study, ^18^F-NaF uptake significantly correlated with serum hsCRP level which is a reliable surrogate biomarker for systemic inflammation in diagnosis of cardiovascular diseases [29]. Furthermore, there was a trend towards higher ^18^F-NaF uptake along with increased LDL and cholesterol levels, which indicates the association between systemical risk markers and the presence of arterial inflammation in the coronaries.

### Limitation

Coronary arterial PET is challenging because of small plaque sizes and cardiac motion. The artifacts related to increased partial volume effects and cardiac motion should not be ignored. Also, due to different image formats of the applied methods, 3D fusion was not possible. Given these limitations, misalignment between PET and CT as well as PET/CT and IVUS cannot be excluded. We also acknowledge the small patient population recruited in this study; we were also lacking histological evidence of coronary plaques to correlate with in vivo imaging as well as motion compensation and partial volume correction for ^18^F-NaF PET/CT imaging of coronary plaque. There were no early atherosclerotic lesions or healthy lesions selected in the IVUS setting, and no invasive morphological evidence was provided to correlate with negative ^18^F-NaF findings. We also could not exclude pharmaceutical effects on the imaging data.

## Conclusion

For the global evaluation of atherosclerotic plaques, the combination of ^18^F-NaF PET and anatomical CT might be employed to provide morphological information about plaque burden, as well as pathophysiological osteogenesis. In clinical practice, ^18^F-NaF PET/CT showed great potential as an adjunctive noninvasive imaging tool to coronary catheterization imaging for risk stratification and guiding therapy.

## Electronic supplementary material

ESM 1 (DOC 83 kb)
