# Supplementary material for: Sodium-fluoride PET-CT for the non-invasive evaluation of coronary plaques in symptomatic patients with coronary artery disease: a cross-correlation study with intravascular ultrasound
Source: Eur J Nucl Med Mol Imaging. 2018 Aug 31;45(12):2181–9. doi: 10.1007/s00259-018-4122-0 (PMC6182395; doi:10.1007/s00259-018-4122-0)
Supplement: Supplementary file 1 — (DOC 83 kb) [file 259_2018_4122_MOESM1_ESM.doc]

Supplemental table 1: All lesioned TBR_max_(^18^F-NaF), Agatston scores, IVUS categorization.

| No. of lesions | TBR_max_ (^18^F-NaF) | Agatston Scores | IVUS category of coronary plaque |
| --- | --- | --- | --- |
| 1 | 0.76 | 10 | thick-cap fibroatheroma with dense necrotic tissue |
| 2 | 0.85 | 31 | thick-cap fibroatheroma with dense necrotic tissue |
| 3 | 1.04 | 331 | thick-cap fibroatheroma with dense necrotic tissue |
| 4 | 1.04 | 1 | thick-cap fibroatheroma with dense necrotic tissue |
| 5 | 1.06 | 6 | thick-cap fibroatheroma with dense necrotic tissue |
| 6 | 1.07 | 0 | thick-cap fibroatheroma with dense necrotic tissue |
| 7 | 1.11 | 190 | thick-cap fibroatheroma with dense necrotic tissue |
| 8 | 1.15 | 0 | thick-cap fibroatheroma with dense necrotic tissue |
| 9 | 1.18 | 295 | thick-cap fibroatheroma with dense necrotic tissue |
| 10 | 1.22 | 0 | thick-cap fibroatheroma with dense necrotic tissue |
| 11 | 1.23 | 0 | thick-cap fibroatheroma with dense necrotic tissue |
| 12 | 1.25 | 6 | thick-cap fibroatheroma with dense necrotic tissue |
| 13 | 1.27 | 5 | thick-cap fibroatheroma with dense necrotic tissue |
| 14 | 1.31 | 41 | thick-cap fibroatheroma with dense necrotic tissue |
| 15 | 1.37 | 142 | thick-cap fibroatheroma with dense necrotic tissue |
| 16 | 1.37 | 1 | thick-cap fibroatheroma with dense necrotic tissue |
| 17 | 1.4 | 0 | thick-cap fibroatheroma with dense necrotic tissue |
| 18 | 1.42 | 186 | thick-cap fibroatheroma with dense necrotic tissue |
| 19 | 1.63 | 15 | thick-cap fibroatheroma with dense necrotic tissue |
| 20 | 1.65 | 15 | thick-cap fibroatheroma with dense necrotic tissue |
| 21 | 2.61 | 1 | thick-cap fibroatheroma with dense necrotic tissue |
| 22 | 0.99 | 162 | thin-cap fibroatheroma with dense necrotic tissue |
| 23 | 1.01 | 224 | thin-cap fibroatheroma with dense necrotic tissue |
| 24 | 1.09 | 341 | thin-cap fibroatheroma with dense necrotic tissue |
| 25 | 1.13 | 0 | thin-cap fibroatheroma with dense necrotic tissue |
| 26 | 1.19 | 66 | thin-cap fibroatheroma with dense necrotic tissue |
| 27 | 1.2 | 0 | thin-cap fibroatheroma with dense necrotic tissue |
| 28 | 1.2 | 90 | thin-cap fibroatheroma with dense necrotic tissue |
| 29 | 1.22 | 109 | thin-cap fibroatheroma with dense necrotic tissue |
| 30 | 1.28 | 5 | thin-cap fibroatheroma with dense necrotic tissue |
| 31 | 1.29 | 338 | thin-cap fibroatheroma with dense necrotic tissue |
| 32 | 1.3 | 94 | thin-cap fibroatheroma with dense necrotic tissue |
| 33 | 1.31 | 79 | thin-cap fibroatheroma with dense necrotic tissue |
| 34 | 1.32 | 42 | thin-cap fibroatheroma with dense necrotic tissue |
| 35 | 1.33 | 75 | thin-cap fibroatheroma with dense necrotic tissue |
| 36 | 1.35 | 148 | thin-cap fibroatheroma with dense necrotic tissue |
| 37 | 1.37 | 290 | thin-cap fibroatheroma with dense necrotic tissue |
| 38 | 1.38 | 64 | thin-cap fibroatheroma with dense necrotic tissue |
| 39 | 1.39 | 43 | thin-cap fibroatheroma with dense necrotic tissue |
| 40 | 1.42 | 0 | thin-cap fibroatheroma with dense necrotic tissue |
| 41 | 1.43 | 5 | thin-cap fibroatheroma with dense necrotic tissue |
| 42 | 1.77 | 220 | thin-cap fibroatheroma with dense necrotic tissue |
| 43 | 2.07 | 0 | thin-cap fibroatheroma with dense necrotic tissue |
| 44 | 0.67 | 5 | fibrotic plaque poor necrotic tissue |
| 45 | 0.68 | 0 | fibrotic plaque poor necrotic tissue |
| 46 | 0.73 | 0 | fibrotic plaque poor necrotic tissue |
| 47 | 0.85 | 113 | fibrotic plaque poor necrotic tissue |
| 48 | 0.85 | 338 | fibrotic plaque poor necrotic tissue |
| 49 | 0.89 | 47 | fibrotic plaque poor necrotic tissue |
| 50 | 0.9 | 3 | fibrotic plaque poor necrotic tissue |
| 51 | 0.93 | 1 | fibrotic plaque poor necrotic tissue |
| 52 | 0.95 | 119 | fibrotic plaque poor necrotic tissue |
| 53 | 0.97 | 0 | fibrotic plaque poor necrotic tissue |
| 54 | 0.98 | 29 | fibrotic plaque poor necrotic tissue |
| 55 | 0.99 | 0 | fibrotic plaque poor necrotic tissue |
| 56 | 1.01 | 0 | fibrotic plaque poor necrotic tissue |
| 57 | 1.09 | 0 | fibrotic plaque poor necrotic tissue |
| 58 | 1.16 | 0 | fibrotic plaque poor necrotic tissue |
| 59 | 1.25 | 64 | fibrotic plaque poor necrotic tissue |
| 60 | 1.3 | 0 | fibrotic plaque poor necrotic tissue |
| 61 | 1.32 | 94 | fibrotic plaque poor necrotic tissue |
| 62 | 1.01 | 431 | fibrocalcific plaque with intensive calcification |
| 63 | 1.22 | 849 | fibrocalcific plaque with intensive calcification |
| 64 | 1.24 | 374 | fibrocalcific plaque with intensive calcification |
| 65 | 1.31 | 1016 | fibrocalcific plaque with intensive calcification |
| 66 | 1.48 | 1553 | fibrocalcific plaque with intensive calcification |
| 67 | 1.56 | 892 | fibrocalcific plaque with intensive calcification |
| 68 | 1.65 | 504 | fibrocalcific plaque with intensive calcification |
| 69 | 1.88 | 371 | fibrocalcific plaque with intensive calcification |
